# The Effect of the Ratio of Butylene Succinate and Dilinoleic Diol in Their Copolyester (PBS-DLS) on the Physicochemical Properties and Biofilm Formation

**DOI:** 10.3390/molecules30061387

**Published:** 2025-03-20

**Authors:** Szymon Macieja, Agnieszka Piegat, Małgorzata Mizielińska, Nina Stefaniak, Mirosława El Fray, Artur Bartkowiak, Magdalena Zdanowicz

**Affiliations:** 1 Center of Bioimmobilisation and Innovative Packaging Materials, Faculty of Food Sciences and Fisheries, West Pomeranian University of Technology Szczecin, Janickiego 35, 71-270 Szczecin, Poland; szymon.macieja@zut.edu.pl (S.M.); mmizielinska@zut.edu.pl (M.M.); artur-bartkowiak@zut.edu.pl (A.B.); 2 Department of Polymer and Biomaterials Science, Faculty of Chemical Technology and Engineering, West Pomeranian University of Technology in Szczecin, Al. Piastow 45, 71-311 Szczecin, Poland; nina.kantor-malujdy@zut.edu.pl (N.S.); mirfray@zut.edu.pl (M.E.F.)

**Keywords:** antibiofilm properties, copolymers, dilinoleic diol, poly(butylene succinate–dilinoleic succinate) (PBS–DLS)

## Abstract

Biofilm-forming microorganisms pose a severe threat in the food and medical industries, among others. In this paper, the research materials were poly(butylene succinate–dilinoleic succinate) (PBS–DLS) copolymers with variable hard and soft segment weight ratios (90:10, 70:30, and 50:50). Polymeric films were prepared by the solvent casting method. Selected physicochemical properties and the tendency to form biofilm on the polymer surface were investigated. As the amount of DLS soft segments in the polymer matrix increased, changes in the FTIR–ATR spectra (signal intensity), surface (SEM), and phase transition (DSC) were observed. The higher the content of the DLS segment, the lower the transition temperatures and the smoother the film’s surface. These factors resulted in a significant reduction in the amount of biofilm formed on the material’s surface and a decrease in the metabolic activity of microorganisms present in the biofilm and SEM micrographs. The obtained PBS–DLS films have great potential in the food and medical packaging industries.

## 1. Introduction

Biofilms, or multicellular communities of microorganisms, threaten and cause harm in many areas of daily life, including the food industry and medicine. Microorganisms encase themselves in envelopes of polysaccharides, proteins, nucleic acids, and lipids that provide them with attachment to surfaces and protection from external agents [1]. Thanks to the formation of biofilms, these microorganisms are more resistant (compared to planktonic cells) to UV radiation, temperature extremes, pH changes, ambient salinity, high pressure, oligotrophic conditions, and even the effects of antibiotics [2]. It has been reported that biofilm microorganisms can withstand antibiotic concentrations up to 1000 times higher than planktonic cells [3,4]. Their strong adhesion to surfaces and resistance to external factors makes them difficult to remove [5].

The mechanism of biofilm formation that is most often presented has been developed from studies on *Pseudomonas aeruginosa*. It consists of five phases, each of which differs from the others regarding gene expression and proteins produced. At the beginning, a reversible attachment is observed. A planktonic cell (or group of cells) attaches to the surface with one pole. Reattachment of the cell can then occur. When the cell attaches itself to the surface with its longitudinal axis, the next phase occurs, thus leading to irreversible attachment. This is when gene expression changes, antimicrobial resistance increases, and the production of cell-surface attachment proteins begins. This is followed by maturation I, where the biofilm reaches a layer of several cell thicknesses and surrounds itself with an extracellular matrix. The biofilm growth process continues until the microcolony formation stage is reached (maturation II). Finally, the final stage—dispersion—occurs, where some cells are released from the biofilm volume [6]. However, as noted by Sauer et al. [6], the described model of biofilm formation was made based on observations for *P. aeruginosa* and should not be universally applied to all microorganisms. Instead, they propose a three-phase model: aggregation, growth, and disaggregation [6].

Biofilm-forming microorganisms are a severe threat in medicine. It is estimated that they are responsible for more than 80% of human infections. They are a crucial problem during burns, chronic wounds, and lung infections and are often linked to the need to connect patients to medical equipment or catheterization [7]. The problem is serious because, as already mentioned, microorganisms in biofilms are up to 1000 times more resistant to antibiotics [3,4] and other external agents [2]. This creates the need to implement more prolonged treatment with higher doses of drugs or to change the approach to treating the infection. It also implies a worsening of patients’ psychological and physical condition and a more significant financial and personnel burden on health care. Ulcerative infections of the diabetic foot caused by biofilms alone generate considerable costs to the health service and often end up requiring amputation of the limb [8]. The microorganisms most commonly associated with biofilm infections in medicine include *Escherichia coli*, *Pseudomonas aeruginosa*, *Klebsiella pneumonia*, *Staphylococcus aureus*, *Candida* spp., *Acinetobacter baumannii*, *Bacillus cereus*, and *Enterococcus faecalis* [9].

Production lines in the food industry create a favorable environment for the formation and development of biofilms due to their complexity and length, which creates opportunities for the deposition of biofilm-forming microorganisms on the surfaces of equipment and transport lines. Moreover, the amount of food passing through them and the substrates available for microbial growth in them are other factors favoring the proliferation of microorganisms and the development of biofilms [10]. These factors, namely surface properties, nutrient content, environmental conditions, and the presence of specific species and strains of microorganisms, affect the dynamics of biofilm development in the food industry [11,12,13,14,15]. Factors related to a given surface that have the most significant impact on cell adhesion and biofilm formation are hydrophobicity, electrostatic charge, and roughness [11,16]. In addition to the obvious consequences of biofilms in the food industry in the form of spoilage of food goods and the risk of consumer poisoning, economic losses cannot be forgotten. Contaminated food falls out of circulation, generating food waste, and biofilms themselves contribute to inferior heat transport, increased resistance to fluid flow, mechanical blockage of systems, and accelerated surface corrosion [17]. The most common microorganisms posing challenges in the food industry are *Bacillus cereus*, *Campylobacter jejuni*, *Escherichia coli*, *Listeria monocytogenes*, *Salmonella enterica*, *Staphylococcus aureus*, and *Pseudomonas* spp. [18].

However, it is essential to remember that biofilms also have another side of the coin, and in some aspects, we have learned to use them to our advantage. An example is biofilm reactors, in which microorganism cells are deposited in the form of a biofilm on supporting materials. These are used in wastewater treatment (by decomposing organic compounds). Using biofilms on support materials facilitates the separation of the liquid phase from the solid phase, providing better economic performance [19,20]. In addition, biofilms and their products have been successfully used to decompose organic environmental contaminants of soil [21]. Despite the extensive research on biofilm formation and its impact in various fields, little attention has been given to the interplay between polymer surface modifications and microbial colonization dynamics. While many studies have explored the antibacterial effects of active additives in polymers, there is limited knowledge regarding how modifications at the molecular level, such as altering the polymer backbone structure with specific monomers, influence biofilm development.

As previously described, bacteria’s proliferation and biofilm formation represent a substantial problem across diverse domains encompassing medical and food-related sectors. Beyond direct bacterial colonization on tissues or food products, potential adherence to proximal surfaces exists. Within medical contexts, susceptible materials include surfaces of catheters, implants, and assorted medical apparatus [22,23,24]. In food production, the primary concern revolves around the predisposition of packaging materials to bacterial colonization. Bacterial colonization is facilitated in both scenarios by a moist environment and ready nutrient accessibility.

In this study, we address this gap by investigating poly(butylene succinate–dilinoleic succinate) (PBS–DLS) copolymers, which incorporate fatty acid-derived monomers, potentially influencing microbial attachment and biofilm formation due to their inherent antimicrobial properties. Unlike previous studies focused on adding antimicrobial agents to polymers, our approach examines how structural modifications at the polymer backbone level alter biofilm susceptibility. Aliphatic polyesters have gained particular attention among polymers used as biomaterials and packaging materials. Most are characterized by appropriate mechanical and thermal properties, ease of processing, and susceptibility to degradation in various environmental conditions [25,26]. One of the representatives of this constantly developing group of polyesters is poly(butylene succinate) (PBS), which, as both a homopolymer and a copolymer, has been tested in many applications, such as tissue engineering, agriculture, and packaging [27,28]. By systematical evaluation of PBS–DLS copolymers with variable hard-to-soft segment ratios (90:10, 70:30, and 50:50), we aim to uncover how these modifications affect microbial colonization and biofilm formation. Our study is the first to investigate the biofilm resistance of PBS–DLS copolymers, offering insights into the relationship between polymer composition and microbial behavior/adhesion. Also, this polymer has been modified with various additives to improve the antibacterial properties of PBS in different forms. In a paper published by Domínguez-Robles J. et al. [28], PBS was modified by adding lignin via melt extrusion and subsequent injection-molding to obtain PBS–lignin composites tested as materials with antioxidant and antimicrobial potential. Although composites up to 15 wt% were obtained, only 2.5 wt% of lignin was enough to achieve the expected properties [29]. Essential oils, edible gums, and free fatty acids were also tested as antibacterial components of PBS electrospun fibers. Additive PBS blends showed antimicrobial behavior against *S. aureus*, *E. hirae*, *S. pyogenes*, and *P. aeruginosa* [30]. Several attempts were made to achieve antimicrobial properties in PBS modification for food packaging applications. Mohamad et al. prepared PBS-based films with essential oils (thymol, kesum, and curry) via solvent casting.

Films containing active compounds showed a zone of inhibition against *S. aureus* and improved properties of stored food (e.g., color) [31]. Petchwattana et al. presented an advanced system for bread protection. Namely, a sachet consists of two major parts, i.e., a controlled release part and an active part. The first part was produced from paper coated with ethylene vinyl alcohol, and the second one was an active part made from PBS and a geraniol essential oil blend. The addition of geraniol oil significantly reduced the colonization of *B. cereus* and *E. coli* strains and influenced vapor transmission [32]. Apart from introducing active ingredients to the polymer matrix, physical methods could be used to control the surface properties of PBS. As presented by Pedroni, modification of the PBS surface by a low-pressure plasma oxygen etching resulted in a nanotextured surface that additionally could be modified by a SiOx nanolayer deposited by plasma-enhanced chemical vapor deposition (PECVD). Both texturing and coating processes strongly influence the antibacterial properties tested against *E. coli* and *S. aureus*, simultaneously affecting oxygen diffusion permeability coefficients and the oxygen transmission rate [33].

**Figure 1:**
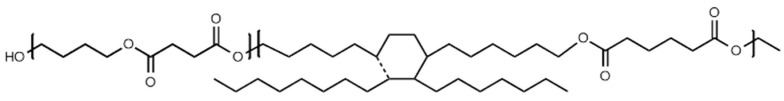
Chemical structure of PBS–DLS copolymers.

Many active agents like essential oils are fragrant and tend to migrate (which can affect the organoleptic properties of packed food). Thus, modification of the polymer by introducing the components into the polymer chain may be one of the resolutions. In the present study, we investigated poly(butylene succinate) copolymers with dilinoleic diol (DLAOH) in terms of the susceptibility to biofilm formation on polymeric surfaces. For the study, the poly(butylene succinate–dilinoleic succinate) (PBS–DLS) copolymers (chemical structure depicted in Figure 1) were selected with variable hard-to-soft segment ratios, namely 90:10, 70:30, and 50:50.

The motivation to undertake this research was multi-threaded. It resulted from both the chemical structure of copolymers and their potential application areas. The use of fatty acid derivatives as monomers allowed us to expect the influence on bacteria behavior since fatty acids are highly recognized as chemicals with antimicrobial properties [34,35,36,37,38]. Even if the fatty acid activity against microorganisms is highest in the free form, the DLAOH monomers affect the morphology of copolymers and, in consequence, several surface-related properties: roughness, spherulite size, water contact angle, susceptibility to degradation, and protein absorption [39,40,41,42]. Changes in bulk properties of polymers modified with DLAOH (thermal, mechanical, solubility in organic solvents, oxygen transmission, etc.) are currently allowed for these types of materials in applications such as packaging materials and materials for tissue engineering or agriculture [43,44,45,46,47]. Due to the above-mentioned potential applications of PBS–DLS copolymers, we decided to investigate materials in contact with various microorganisms to identify their susceptibility to colonization and biofilm formation. For the preliminary tests, polymeric films were obtained via the casting method, and basic physicochemical characterization was performed. The obtained polymeric films were placed in a liquid culture of microorganisms to grow biofilms on their surface. The amount of biofilm formation, metabolic activity of microorganisms on the surface of the films, IR spectroscopic analyses, and SEM images of the films’ surfaces were examined to evaluate the differences in antimicrobial properties between the film variants.

## 2. Results and Discussion

### 2.1. Polymer Synthesis and Characterization of Physicochemical Properties

**Figure 2:**
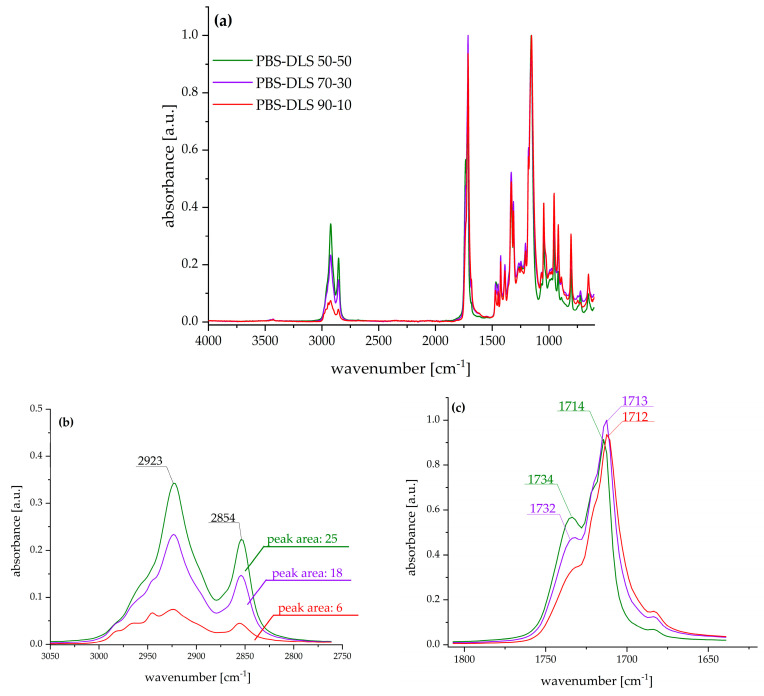
FTIR–ATR spectra of PBS–DLS films: (**a**) complete spectra range; (**b**) region corresponding to aliphatic soft segment backbone; (**c**) region corresponding to the ester carbonyl group in the polymer backbone.

The appearance of the casted films is presented in the Supplementary Materials (SM) in Figure S1. To demonstrate the differences in the chemical structure of PBS–DLS copolymers, FTIR–ATR analysis was performed. Figure 2a presents spectra in the complete scanning range, and the following characteristic bands were observed: 3000–2800 cm^−1^ related to –CH_3_ and –CH_2_– in aliphatic compounds (C-H stretch), 1770–1670 cm^−1^ related to the carbonyl group (C=O), 1470–1300 cm^−1^ –CH_3_ and –CH_2_– in aliphatic compounds, 1230–1100 cm^−1^ C-O-C in esters, and 1047 cm^−1^ CH_2_-O-H (C-O stretch).

The most significant changes were observed in the region of 3000–2800 cm^−1^, characteristic of methyl groups from aliphatic chains, and in the region of 1770–1670 cm^−1^ assigned to a carbonyl group. With the increased weight fraction of soft segments composed of dilinoleic diol intensity of C-H antisymmetric (2923 cm^−1^) and symmetric (2854 cm^−1^) the stretching bands increased and, in consequence, the area under the corresponding peaks also increased (as pointed out in Figure 2b). Furthermore, the increased amount of DLAOH influenced the shape of the carbonyl band—the higher the amount of fatty acid diol, the higher the intensity of the band, with a maximum at ~1732 cm^−1^, typical for esters, and at ~1713 cm^−1^, characteristic for carboxylic acids.

**Figure 3:**
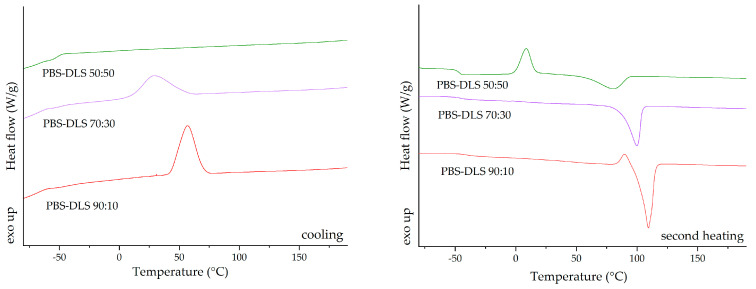
DSC thermograms for cooling and second heating run.

The differential scanning calorimetry (DSC) analysis was repeated for the new batch of PBS–DLS copolymers, synthesized according to the method described by Stępień et al. (2019) [48], to ensure the reproducibility of the obtained copolyesters. PBS–DLS copolymers with varying compositions indicated distinct thermal properties that could be correlated with their composition ratios. The glass transition temperature (T_g_) of the copolymers showed a trend of increasing values with a higher concentration of the dilinoleic diol-based segment; this was evident because the T_g_ shifted from −41 °C for PBS–DLS 90:10 to −46.9 °C for PBS–DLS 50:50 (Figure 3). This suggests enhanced segmental mobility as the dilinoleic diol content increased. The melting temperature (T_m_) and the enthalpy of melting (ΔH_m_) decreased with the dilinoleic diol content, indicating a less crystalline structure in copolymers with a higher DLS segment content. The cold crystallization temperature (T_cc_) and the enthalpy of cold crystallization (ΔH_cc_) were observed only for the PBS–DLS 50:50 material, suggesting that this composition can further crystallize upon heating, which is indicative of its less ordered structure as initially formed. The DSC analysis of the PBS–DLS copolymers revealed that the ratio of constituents significantly influenced the thermal properties, with higher DLS content leading to lower crystallinity and higher segmental mobility, as reflected in the T_g_, T_m_, and crystallization behavior of the materials. The thermograms for PBS–DLS films are presented in Figure 3 and values of the temperature for phase transition listed in Table 1.

**Figure 4:**
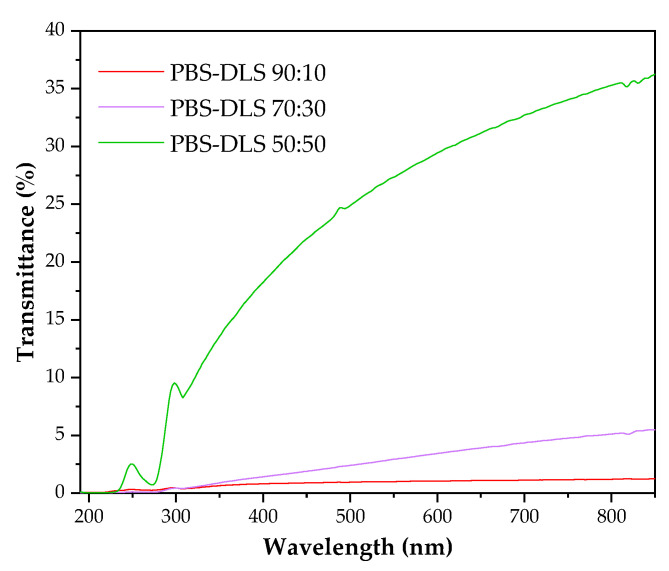
UV-Vis spectra of PBS–DLS films.

Analysis of the wettability of polymer films prepared from PBS–DLS copolymers showed that all materials exhibited water contact angle values in the range of 84–90 degrees (PBS–DLS 90-10 85 ± 0.3; PBS–DLS 70-30 92 ± 4; PBS–DLS 50–50 84 ± 0.34), confirming the hydrophobic character of all surfaces. Changes in the chemical composition of the copolymers did not significantly influence the hydrophobicity between the series, and similar observation was reported by Staniszewski et al. [49] for poly(ethylene terephthalate) modified with linoleic acid dimer. The subsequent critical factor influencing the wettability of polymer surfaces is surface roughness, at both the micro- and the nanoscale. This parameter must be considered in the present discussion. Most polymer modifications are designed to enhance hydrophobicity and the repellence of water molecules. Apart from polymer chemistry, one strategy for regulating this parameter involves incorporating micro- and/or nanotopography on the polymer surface. Puukilainen et al. [50] have presented an efficient method for modifying polyolefins for packaging applications. This method consists of the application of inserts to injection molds, thereby inducing changes in the polymers’ topography. The visual appearance of the films obtained via the casting method was milky and opaque; however, the higher content of the DLS fraction, the more transparent sample there was. Figure 4 shows that PBS–DLS 90:10 and PBS–DLS 70:30 films were barely transparent for visible light and that PBS–DLS 50:50 exhibited transparency (at 750 nm) of 34%. All samples blocked UV radiation, especially UV-C and UV-B.

### 2.2. Biofilm and Microbial Metabolic Activity Evaluation

The abbreviations of the names of microorganisms used to describe the results are EC—*Escherichia coli*, SA—*Staphylococcus aureus*, CA—*Candida albicans*, BA—*Bacillus atrophaeus*, BS—*Bacillus subtilis*, and BC—*Bacillus cereus*.

**Figure 5:**
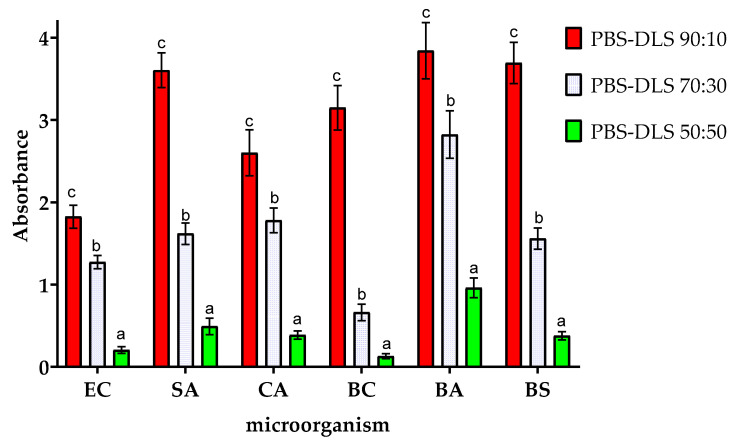
Effect of the ratio of PBS to DLS in the polymer matrix on the amount of biofilm formation by the microorganisms studied. EC—*Escherichia coli*, SA—*Staphylococcus aureus*, CA—*Candida albicans*, BA—*Bacillus atrophaeus*, BS—*Bacillus subtilis*, and BC—*Bacillus cereus.* For each microorganism, bars with letters a–c above are significantly different at *p* < 0.05.

Figure 5 shows the effect of the PBS–DLS segmental composition on the amount of biofilm formed on the surface of the films. As can be seen, the chemical composition of PBS–DLS copolymers significantly affected the susceptibility to biofilm formation on their surface. The differences in the relative amount of biofilm formed on the surface of the tested materials with different PBS–DLS ratios were statistically different (*p* > 0.05). Compared to films with PBS–DLS ratios of 90:10, films with higher DLS content showed lower susceptibility to biofilm formation. The reductions in biofilm formation for PBS–DLS 70:30 and 50:50 films (compared to the 90:10 film) were as follows: 30.2% and 88.8% lower for EC, 55.1% and 86.4% lower for SA, 31.6% and 85.2% lower for CA, 26.8% and 72.3% lower for PA, 79.0% and 95.90% lower for BC, 26.5% and 75.0% lower for BA, and 57.8% and 89.7% lower for BC, respectively. The most significant differences between PBS–DLS 90:10 and PBS–DLS 50:50 films were observed for BC (more than 24-fold reduction in biofilm formation) and BS (nearly 10-fold reduction in biofilm formation).

Domínguez-Robles et al. described research on PBS–lignin composites in the context of potential application in the biomedical industry. They noted that adding 2.5% to 15% lignin reduced *S. aureus* biofilm formation by about 90%. However, adding lignin did not significantly change the wetting angle, and as the lignin content increased, the material’s surface became more uneven [29]. This indicates that, in their case, the obtained antimicrobial properties were due to the antimicrobial effect of lignin, which managed to outweigh the potential deterioration of this parameter caused by changes in surface morphology. Attempts to modify PBS-based films with curcumin and carvacrol also significantly reduced the biofilm formed on the material’s surface. *E. coli*, *S. aureus*, and *C. albicans* were used in this study. The addition of 1 wt% of curcumin and 1 wt% of carvacrol to modify PBS resulted in a significant reduction in *E. coli* biofilm and a reasonable reduction in *C. albicans* biofilm. The weakest antibiofilm effect was observed for *S. aureus*; however, the reduction in the amount of biofilm formed was still statistically significant [51]. In this study, the mechanism behind the antibiofilm properties was not determined. Still, based on the antimicrobial results obtained, it can be suspected that this was the mechanism that reduced the amount of biofilm on the surface of the materials obtained.

**Figure 6:**
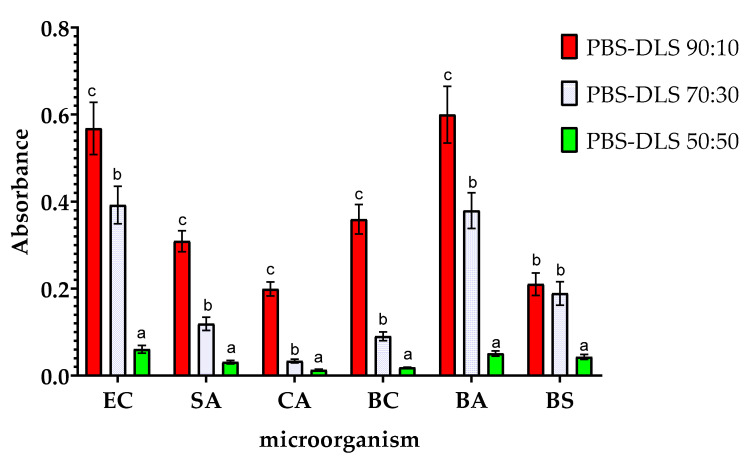
Effect of the ratio of PBS to DLS in the polymer matrix on the metabolic activity of the microorganisms studied. EC—*Escherichia coli*, SA—*Staphylococcus aureus*, CA—*Candida albicans*, BA—*Bacillus atrophaeus*, BS—*Bacillus subtilis*, and BC—*Bacillus cereus.* For each microorganism, bars with letters a–c above are significantly different at *p* < 0.05.

The results of the analysis of the metabolic activity of biofilms obtained on the surfaces of the samples are shown in Figure 6. These results are consistent with those obtained in the study of the amount of biofilm formed, but there are slight differences. Compared to the results for PBS–DLS 90:10 film, samples with a higher DLS ratio showed lower biofilm metabolic activity. The highest degree of reduction in observed metabolic activity was observed for BC (a reduction of nearly 20-fold) and for CA (more than 15-fold). This demonstrates the fact that microorganisms adhered to the surface of the films remain metabolically active. Nevertheless, with an increase in the proportion of DLS in the polymer matrix, a decrease in metabolic activity is observed, which can be associated with the adhesion of fewer microorganism cells to the surface of the films. It is worth noting that although the MTT reduction assay with formazan crystals forming is most often used when studying the metabolic activity of eukaryotic cells, there are several reports on the successful use of this assay also when studying microbial cells. However, the exact mechanism of MTT reduction by microbial cells is not fully understood. The final result of the assay can be influenced by the parameters of the conducted culture (time, pH), the properties of the strain (type of strain, growth phase, multidrug resistance of the strain), or the composition of the culture medium. The final result may be falsely overestimated or underestimated. The results may vary from one trial to another, and the results can only be compared with each other within the same assay [52].

**Figure 7:**
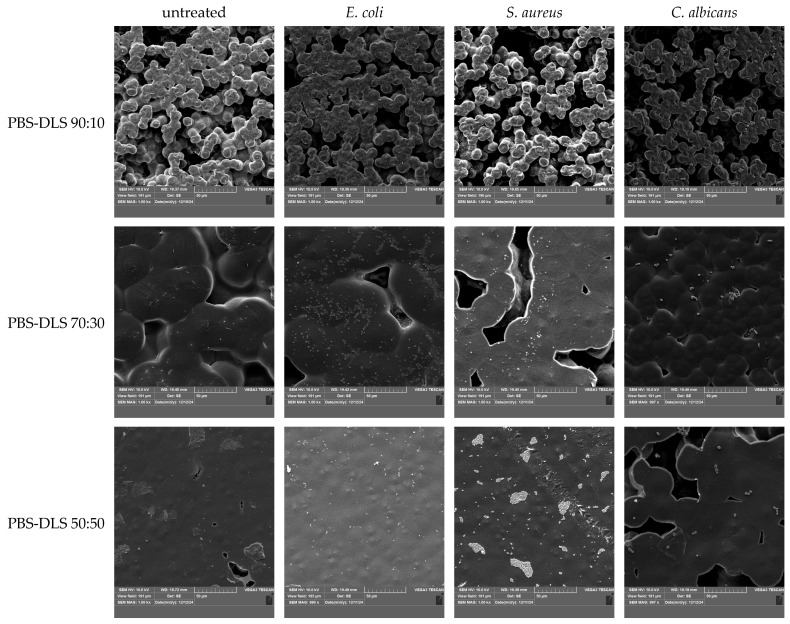
SEM photos of untreated films and films after biofilm cultivation.

The mechanism of interaction between bacteria and polymer surfaces is a complicated process, and its complexity results from many factors characterizing both solid surfaces and the properties of the microorganisms themselves. The most important surface properties of materials include their chemical structure, surface energy, surface charges, wettability, and roughness/topography—all these features are, in turn, a consequence of the material’s chemical structure, behavior in the aqueous environment, and possible surface modifications (physical or chemical) [53,54]. Considering the mechanism of biofilm formation presented earlier, each of these properties affects the adhesion of bacteria and their ability to colonize and develop the EPS network [55,56]. As emphasized in the works, it is difficult to determine one generally applicable mechanism of interactions between bacteria and the polymer surface due to the mutual specificity of both critical elements. The results obtained for the tested copolymers also emphasize the complexity of this process. Data regarding the amount of biofilm produced and its activity indicated that the PBS–DLS 90:10 copolymer was most vulnerable to the adhesion of microorganisms and biofilm formation. However, looking at the changes in the chemical structure of the surface, the most intense changes were observed for microorganisms on the surface of the PBS–DLS 50:50 copolymer. This may suggest a different strength of bacteria’s interaction with other surfaces—as noted in Ref. [57], it is even possible to detach the biofilm if the surface roughness is not appropriate to ensure its strong enough adhesion and could be detached, e.g., by repeated rinsing of biofilms, e.g., to remove free bacterial cells. Figure 7 depicts surface morphology recorded by SEM. It is visible that the DLS content in the copolymer affected this feature. The sample with the lowest DLS content had a porous inhomogeneous microbead structure, similar to pure PBS [58] (first column). The higher the content of DLS, the smoother and more homogenous the morphology was. These results correspond to the DSC results (lower T_g_ and T_m_) (Figure 3, Table 1) and the UV-Vis spectra (Figure 4), where, for a higher content of DLS, higher transparency was obtained. This confirms that introducing DLAOH into polymer acts as an internal plasticizer that facilitates film formation by the casting method.

Due to the aforementioned differences in the surface morphology of the films, there were differences in the biofilm formation of the microorganisms studied (the higher magnitude of the film surface is presented in Figure S3). In the case of the micro-beaded structure of PBS–DLS 90:10 films (average diameters of microbeads forming aggregates—11.51 µm ± 2.49, Figure S4), the microorganisms had a larger specific surface area for colonization. At the same time, they were dispersed both on the surface and in the nooks and crannies of the film. SEM images show only those present on the surface. For PBS–DLS 70:30 samples, an intermediate structure between beaded and smooth (the microbeads were flattened, forming a more integrated structure with “meeting boundaries”) can be observed. The diameter of the beads was greater and the free spaces were smaller, which decreased the surface area for microorganisms. This resulted in more apparent microbial cells on the surface of the film. However, combined with the results of crystal violet staining and the ability to metabolize MTT, it can be concluded that a smaller portion of the cells was present inside the indentations in the structure of the films. For PBS–DLS 50:50 films, the observed surface was even smoother and free spaces were less frequent. As a result, the specific surface area of the film was the smallest. The surface showed single cells of *E. coli* or *C. albicans*. For 50:50 PBS–DLS film with *S. aureus* biofilm, clusters of cells could be seen forming on the surface, characteristic for staphylococci. Based on the SEM image alone, it would seem that colonization by *S. aureus* was the highest for this sample. However, taking into account the smoothest surface and the results of violet staining and metabolic activity, it can be concluded that as a result of the low availability of space on the surface of the films, the transition of cells to the next stage of biofilm production was observable, i.e., intensive cell division and formation of microcolonies. In all other images, the initial stages, that is, adhesion to the surface and cell proliferation, were observed. Taking into account the differences in the film wettability and surface structure, it can be concluded that as the proportion of DLS in the polymer matrix increased, less surface area was available for the colonization and it was harder for microorganisms to adhere to it.

## 3. Materials and Methods

### 3.1. Materials and Reagents

The following reagents were used for polymer synthesis: butanediol (1,4-BD) (Alfa Aesar; Kandel, Germany), dimer linoleic diol (Pripol 2033, Croda International, Gouda, The Netherlands), and dimethyl succinate (Sigma Aldrich, Hamburg, Germany).

Peptone water and MacConkey medium were from Scharlau Chemie (Barcelona, Spain). Cristal violet, 3-(4,5-Dimethyl-2-thiazolyl)-2,5-diphenyl-2H-tetrazolium bromide (MTT), Chapman’s medium, plate count agar, Sabouraud dextrose agar, and tryptic soy broth were from Merck (Dramstadt, Germany). Chloroform, dimethyl sulfoxide, and acetic acid were supplied from Chempur (Piekary Śląskie, Poland). All reagents were of analytical grade.

The microorganisms used to evaluate the films’ antimicrobial properties were obtained from the American Type Culture Collection (ATCC, Manassas, VA, USA). The strains used were *Escherichia coli* ATCC8739, *Staphylococcus aureus* ATCC6538, *Candida albicans* ATCC14053, *Bacillus atrophaeus* ATCC9372, *Bacillus subtilis* ATCC19659, and *Bacillus cereus* ATCC14579.

### 3.2. Synthesis of PBS–DLS

The synthesis of copolyesters was conducted through a two-step melt process. Initially, in the transesterification step dimethyl succinate (ester) and 1,4-butanediol were reacted at 180 °C using a titanium dioxide/silicon dioxide (C-94) catalyst, continuing until 95% of the by-product was collected. Subsequently, dilinoleic diol and a portion of the C-94 catalyst were added. Polycondensation commenced upon reducing the pressure to 0.2 hPa and raising the temperature to 245 °C. The reaction’s progress was monitored by measuring the stirrer’s power consumption in a fully automated polycondensation unit. A comprehensive description of the synthesis process for the PBS–DLS materials can be found in the paper by Stȩpień et al. [48]. The chemical structure of the PBS–DLS copolymers is presented in Figure 1.

### 3.3. Polymeric Film Preparation and Characterization

PBS–DLS copolymers were dissolved in chloroform to obtain 80 mg/mL concentrations. The process was performed for 12 h with magnetic stirring (250 rpm) to ensure complete polymer dissolution. Solutions were cast on Petri dishes (pouring 10 g of solutions per 90 mm diameter dish) and solvent was evaporated for 24 h under a fume hood. The chemical structure of the obtained films was analyzed using a Perkin Elmer Spectrum 100 FTIR spectrophotometer (Waltham, MA, USA) equipped with attenuated total reflectance (ATR). Absorbance spectra in the range of 4000 to 600 cm^−1^ at 1 cm^−1^ resolution (32 scans) were recorded. Before compilation, the spectra in absorbance mode underwent automatic baseline correction in OMNIC 7.3 software. For the detailed analysis, the area under the peak was measured using the “peak area tool” in OMNIC software within the wavenumber range of 3050–2750 cm^−1^.

Thermal analysis of the PBS–DLS copolymers was performed using a TA Instruments DSC Q2500 (New Castle, DE, USA), with a temperature range of –90 to 200 °C and a heating/cooling/heating rate of 10 °C/min.

The water contact angle of films was tested by applying a 2 μL droplet volume of distilled water onto the sample through a KRUSS drop-shape analyzer (Hamburg, Germany). Each sample underwent ten measurements. The testing process was conducted following the BS EN 828:2013 standard [59]. The camera angle was set at +1 degree, and the magnification was 7×. The laboratory temperature was maintained at 23.5 °C. The fitting method chosen for the drop was always “circle.” The recording was set for 20 s, and readings were taken after 5 s.

The UV-Vis spectrophotometry (Thermo Scientific Evolution 220 (Waltham, MA, USA)) was used to study transparency and UV light absorption capacity. The test was performed in the wavelength range of 190–900 nm.

### 3.4. Biofilm and Microbial Metabolic Activity Evaluation

To determine changes in the amount of biofilm formation on the surface of the films under study, at first, 3 mL of TSB was poured into the wells of a 12-well plate. Then, 50 μL of liquid microbial cultures were added at an optical density of 0.5 on the McFarland scale. The UV-sterilized test films (1.5 cm × 1.5 cm) were then added to the wells at a rate of 1 film per well. The plates were placed at 37 °C for a 48 h incubation. Then, using tweezers, the films were gently removed from the wells and rinsed 5 times with saline. Films prepared in this way were either transferred directly to test the amount of biofilm formed and metabolic activity or placed in an incubator at 30 °C to dry for the remaining analyses. All samples (for each film and microorganism) were prepared in 12 replicates.

To test the amount of biofilm formed, we pulled fresh samples from the culture, washed them with saline, and then placed them in the wells of a new 12-well plate. Afterward, we added 2 mL of 0.1% crystal violet solution to each well. After 15 min, the films were pulled out again and washed 3 times with distilled water until the unbound crystal violet was completely rinsed off. The films were then placed in falcon tubes, to which 5 mL each of 30% acetic acid was added to dissolve the dye. Finally, the absorbance of the resulting solutions was measured at 595 nm.

Microbial viability was measured using the MTT assay according to the methodology of Wajs-Bonikowska et al. [60] with slight modifications. Samples obtained from biofilm cultures were immediately given a metabolic activity test. Films were placed individually in the wells of a new 12-well plate, and then 3 mL of fresh TSB and 200 μL of MTT solution were added to the wells and left at 37 °C for 4 h. After this time, the films and themedium were removed from the wells with a pipette, leaving only the formazan crystal at the bottom, and 1 mL of DMSO was added to the wells. Absorbance at 570 nm was then measured.

The films before and after biofilm cultivation (representatives of Gram-negative, Gram-positive, and fungi—*Escherichia coli*, *Staphylococcus aureus*, and *Candida albicans*, respectively) (Section 3.4) were examined using a scanning electron microscope (SEM). The samples were attached to the pin stubs and coated with a thin layer of gold in a sputter coater at 24 °C (Quorum Technologies Q150R S, Laughton, East Sussex, UK). Then, SEM micrographs were obtained using a Vega 3 LMU microscope (Tescan, Brno-Kohoutovice, Czech Republic). The microscopic analysis used a tungsten filament with an accelerating voltage of 10 kV.

## 4. Conclusions

This work, for the first time, presents the influence of the plant-based dimer of linoleic acid derivative in PBS–DLS copolyester films. For the preliminary tests, materials were obtained via the casting method. Additionally, the basic physicochemical properties of the films were investigated. DSC results revealed that DLS fraction acted as an internal plasticizer, leading to lower T_g_, T_m_, and ΔH_m_ of the materials. Moreover, the increase in the soft segment fraction in the copolymer led to better film formation and higher transparency of the films. The present work confirms the possibility of modifying the PBS polymer matrix using dilinoleic diol to obtain biofilm inhibition properties. This effect was dependent on the PBS–DLS ratio and increased with increasing amounts of DLS fraction (up to a level of 50:50, for which a reduction of about 90% was achieved compared to the PBS 90:10 DLS sample). According to the analysis, this may be influenced by many factors, including changes in surface roughness and wettability. However, the introduction of hydrophobic side chains into the structure of PBS–DLS copolymers appears to play a fundamental role in inducing a contact-killing mechanism for bacteria that is similar to that of antimicrobial peptides. Potentially, such films could be used in the packaging of food products or medical instruments. Nevertheless, further analysis is needed to determine the functional properties of the obtained bio-based copolyesters, including other methods of processing film preparation, like thermoforming or cast extrusion, for potential packaging materials.

## Figures and Tables

**Table 1:** DSC results.

Sample	*T_g_* [°C]	*T_m_* [°C]	ΔH_m_ [J/g]	*T_c_* [°C]	*ΔH_c_* [J/g]	*T_cc_* [°C]	Δ*H_cc_* [J/g]
PBS–DLS 90:10	−41.1	109.3	75.4	56.8	59.4	-	-
PBS–DLS 70:30	−46.5	99.4	47.5	28.5	46.2	-	-
PBS–DLS 50:50	−47.0	80.6	31.2	-	-	9.1	26.7

## Data Availability

The original contributions presented in this study are included in the article/Supplementary Materials. Further inquiries can be directed to the corresponding authors.

## Supplementary Materials

The following supporting information can be downloaded at: https://www.mdpi.com/article/10.3390/molecules30061387/s1, Figure S1: Photographs of the films. Figure S2: Exemplar FTIR-ATR spectra of the films before and after Escherichia coli biofilm cultivation. Figure S3: SEM graphs of untreated films and films after biofilm cultivation—magnification ×3000. Figure S4: Micrograph of PBS–DLS 90:10 film and microbead diameters.

## Abbreviations

The following abbreviations are used in this manuscript:

BA	*Bacillus atrophaeus*
BC	*Bacillus cereus*
BS	*Bacillus subtilis*
CA	*Candida albicans*
DLAOH	Dilinoleic diol
DLS	Dilinoleic succinate
DSC	Differential scanning calorimetry
EC	*Escherichia coli*
FTIR	Fourier transform infrared spectroscopy
MTT	(3-(4,5-Dimethylthiazolyl-2)-2,5-diphenyltetrazolium bromide)
PBS	Poly(butylene succinate)
PBS-DLS	Poly(butylene succinate-co-dilinoleic succinate)
SA	*Staphylococcus aureus*
SEM	Scanning electron microscopy
