# Supplementary material for: The Effect of the Ratio of Butylene Succinate and Dilinoleic Diol in Their Copolyester (PBS-DLS) on the Physicochemical Properties and Biofilm Formation
Source: Molecules. 2025 Mar 20;30(6):1387. doi: 10.3390/molecules30061387 (PMC11944411; doi:10.3390/molecules30061387)
Supplement: Supplementary file 1 [file molecules-30-01387-s001.zip › molecules-3486966-supplementary.pdf]

SUPPLEMENTARY MATERIALS

The effect of the ratio of butylene succinate and dilinoleic diol in their copolyester (PBS-DLS) on the physicochemical properties and biofilm formation

Szymon Macieja[1], Agnieszka Piegat[2*], Małgorzata Mizielińska[1], Nina Stefaniak[2], Mirosława El Fray[2], Artur Bartkowiak[1], Magdalena Zdanowicz[1*]

[1] Center of Bioimmobilisation and Innovative Packaging Materials, Faculty of Food Sciences and Fisheries, West Pomeranian University of Technology Szczecin, Janickiego 35, 71-270 Szczecin, Poland
[2] Department of Polymer and Biomaterials Science, Faculty of Chemical Technology and Engineering, West Pomeranian University of Technology in Szczecin, Al. Piastow 45, 71-311 Szczecin, Poland
* Correspondence: mzdanowicz@zut.edu.pl; Agnieszka.Piegat@zut.edu.pl

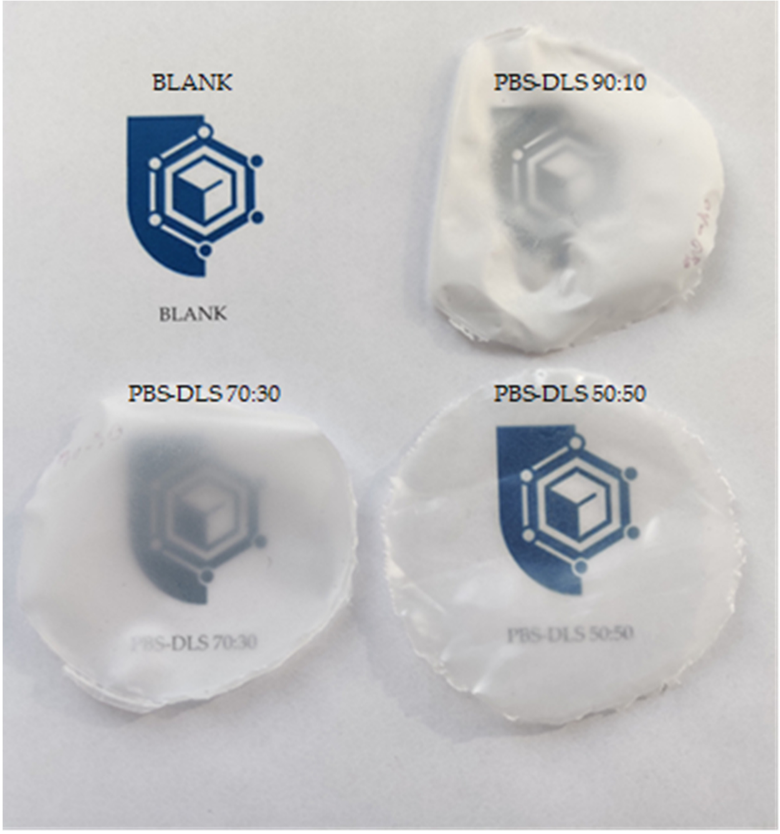

Figure S1. Photographs of the films.

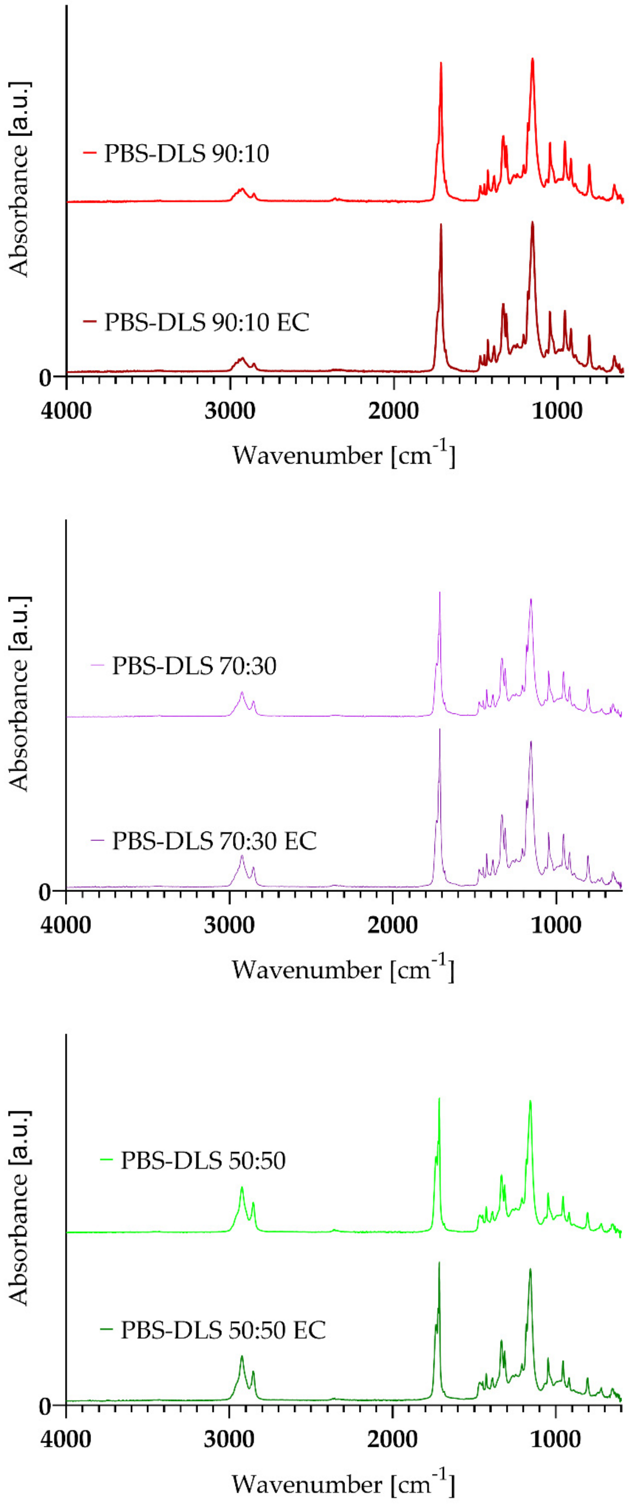

Figure S2. Exemplar FTIR-ATR spectra of the films before and after *Escherichia coli* biofilm cultivation.

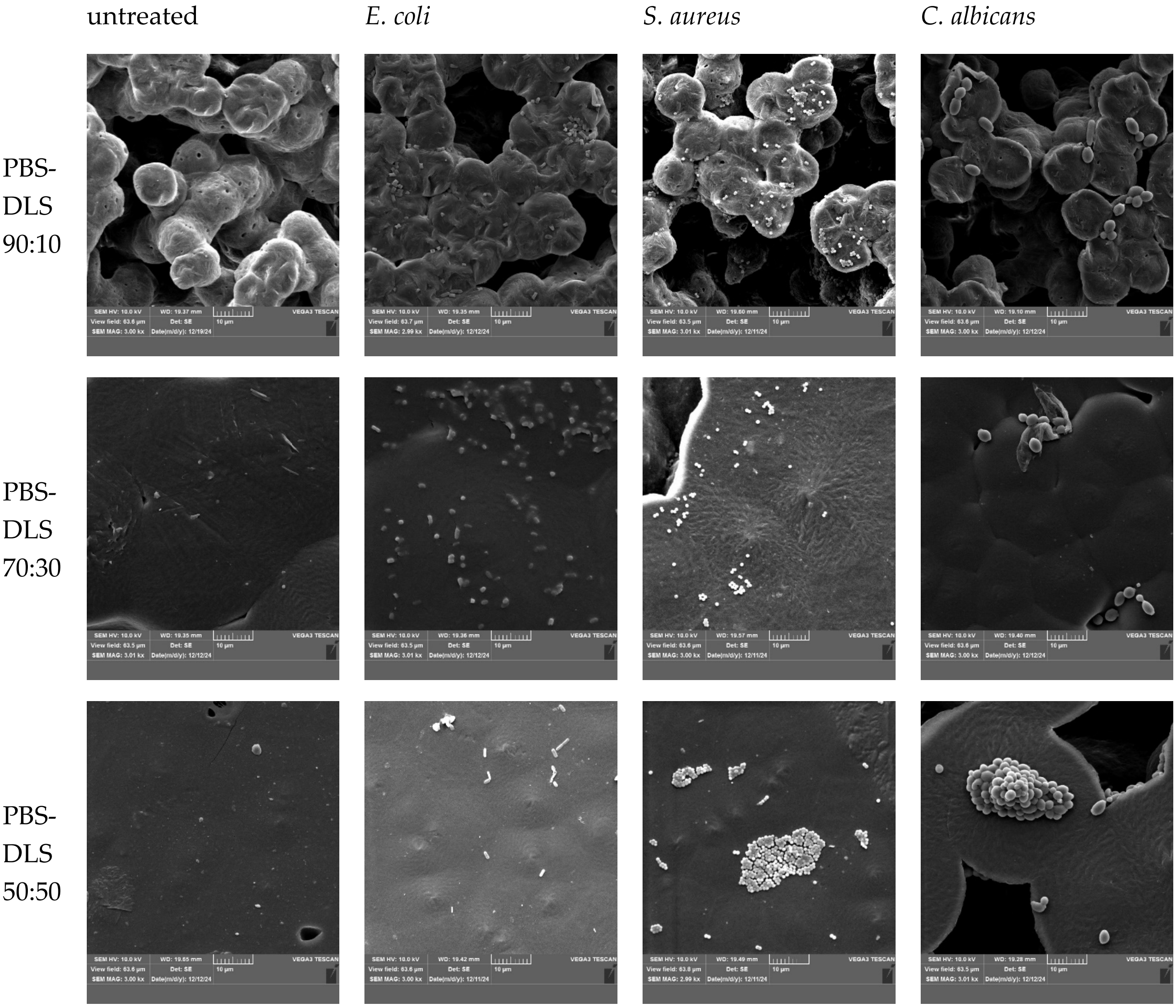

Figure S3. SEM graphs of untreated films and films after biofilm cultivation – magnification ×3000.

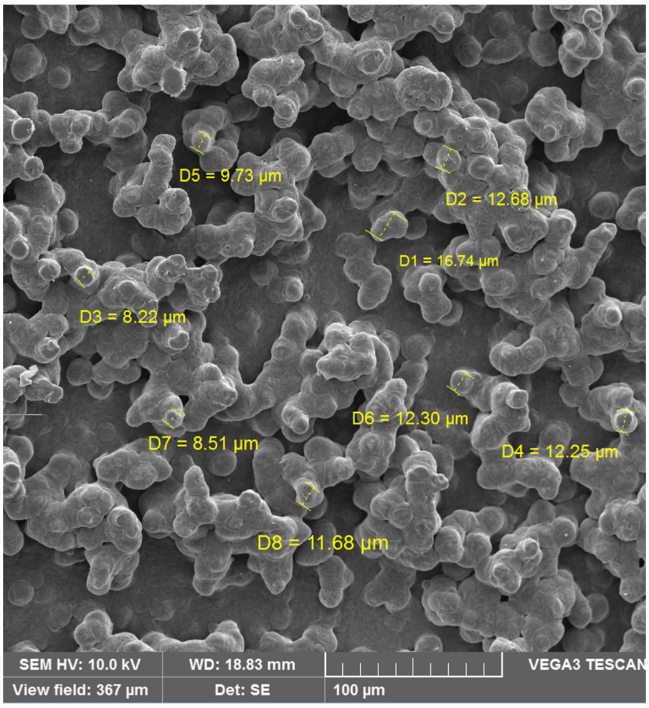

Figure S4. Micrograph of PBS-DLS 90:10 film and microbeads diameters.
